# Emotional learning promotes perceptual predictions by remodeling stimulus representation in visual cortex

**DOI:** 10.1038/s41598-019-52615-6

**Published:** 2019-11-14

**Authors:** E. Meaux, V. Sterpenich, P. Vuilleumier

**Affiliations:** 1 Laboratory for Behavioral Neurology and Imaging of Cognition, Department of Neuroscience, University of Geneva, 1202 Geneva, Switzerland; 2 Laboratory of Cognitive Neurosciences (LNC²), UMR INSERM U960, Ecole Normale Supérieure, PSL Research University, 75005 Paris, France; 3 Department of Clinical Neurology, University Hospital of Geneva, 1206 Geneva, Switzerland; 4 Swiss Center for Affective Sciences, University of Geneva, 1202 Geneva, Switzerland

**Keywords:** Perception, Amygdala

## Abstract

Emotions exert powerful effects on perception and memory, notably by modulating activity in sensory cortices so as to capture attention. Here, we examine whether emotional significance acquired by a visual stimulus can also change its cortical representation by linking neuronal populations coding for different memorized versions of the same stimulus, a mechanism that would facilitate recognition across different appearances. Using fMRI, we show that after pairing a given face with threat through conditioning, viewing this face activates the representation of another viewpoint of the same person, which itself was never conditioned, leading to robust repetition-priming across viewpoints in the ventral visual stream (including medial fusiform, lateral occipital, and anterior temporal cortex). We also observed a functional-anatomical segregation for coding view-invariant and view-specific identity information. These results indicate emotional signals may induce plasticity of stimulus representations in visual cortex, serving to generate new sensory predictions about different appearances of threat-associated stimuli.

## Introduction

Emotions exert powerful effects on perception and cognition but their exact mechanisms remain unresolved. Among others, emotions can guide selective attention by modulating sensory pathways through modulatory feedback from the amygdala, serving to amplify perceptual processing of behaviorally relevant events^1^. However, it is unknown whether such emotion-driven influences on cortical areas can also promote neural plasticity within perceptual pathways and thus produce longer-lasting changes to enable more efficient recognition in subsequent encounters. Emotional effects on learning and memory have mainly been studied in relation to stimulus associations formed in the amygdala or episodic traces formed in the hippocampus, but it is also possible that emotion signals can directly impact on the cortical representations of behaviorally relevant stimuli. For instance, in humans, fear conditioning for a particular visual object (e.g., dog) may enhance subsequent brain responses to objects from the same semantic category (e.g., other dogs or animals), an effect suggesting that emotions strengthen memory associations at the semantic level^2^ and possibly accounting for fear generalization in certain pathological conditions^3^. However, these observations do not establish whether cortical plasticity occurs at the level of a specific stimulus representation based on previous emotional experiences with this stimulus.

One possible impact of emotion-driven plasticity in the cortex might be to promote associative processes linking different sensory features of a threat cue, and thus contribute to generate new perceptual predictions about behaviorally relevant information. Because in the real world stimuli rarely occur twice under the exact same conditions, efficient recognition should enable the brain to identify a source of threat even when its sensory appearance vary. However, cortical neurons are usually highly selective to specific sensory features (e.g. shape or color), and the representation of a particular meaningful object must integrate distinct neuron populations that code for different features of this object (e.g., allowing for some variations in shape or color)^4^. Here, we therefore investigated whether emotion signals can promote such integration between neuronal populations encoding distinct features of the same stimulus. Specifically, we tested if the learned emotional significance of a visual stimulus would induce lasting changes in its cortical representation, and thus enable its recognition across different appearances.

Face recognition is indeed a remarkable capacity allowing us to identify an individual even with changing views, sometimes after a single encounter. This capacity is subserved by dedicated brain networks, including the fusiform and occipital face areas (FFA and OFA). However, the nature of face identity (ID) representation in these areas remains poorly understood. It is controversial whether the visual cortex forms view-invariant representations of familiar faces^5^ or stores image-based representations from encountered views only^6^. Several neuroimaging studies tested for repetition priming with different viewpoints of the same face and found no repetition effects in FFA across viewpoints^7–10^, in keeping with view-based representations. However, view-independent representations might be formed in other regions^11,12^.

Importantly, some computational models proposed that view-independent recognition might operate by linking different view-specific representations though associative learning processes^13,14^. Thus, seeing one particular stimulus view may trigger the retrieval of related information and generate sensory predictions about other views or other features associated with this stimulus. In other words, while face-recognition neurons would code for particular views of particular faces, view-invariant recognition could emerge by linking different views acquired during past exposures or by constructing intermediate views through interpolation mechanisms processes^13^. More broadly, such mechanisms would accord with the notion that perception is an active process combining sensory inputs with stored knowledge, forming predictive models of the world that may dynamically be sharpened by new experiences^15,16^. In this predictive coding framework, the representation of face identity might emerge from the ability to predict different views of the same face, through enhanced connections between different neuronal populations in visual cortex.

**Figure 1:**
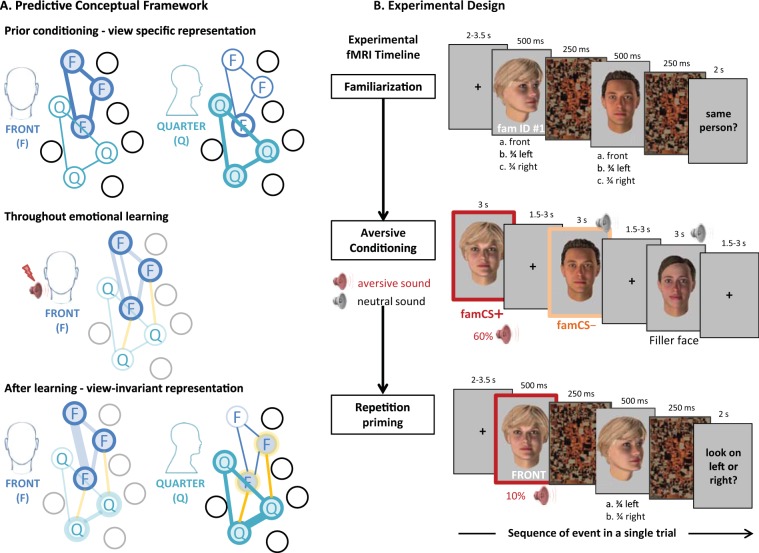
**A.** **Predictive conceptual framework of emotion-driven plasticity in cortical representations of face ID**. **Prior to conditioning**, we expect no overlapping activation of neurons coding for front (F) and quarter (Q) views among the cortical population. Each presentation of a face elicits a view specific representation. **Throughout emotional signals** mediated by amygdala feedback, we hypothesize that the representation of a face in front-view will not only be strengthened after being paired with threat (blue doted line), but also “enlarged” to become associated with another view (e.g. quarter) of the same face (yellow dotted line). **After emotion learning**, we predict that these new connections will lead to overlapping activation of F and Q neurons coding for the same face, leading to the emergence of face ID priming across different viewpoints. *N*.*B*. *Each circle corresponds to one hypothetical neuron and lines represent connections between them within a cortical population*. *Filled circles indicate active neurons whereas empty circles indicate inactive neurons in response to either F or Q face presentation*. **B. Experimental Design. Familiarization session**: Full-front and ¾-views (left or right) images of two neutral faces were presented in a rapid succession, always separated by a scrambled visual mask. Participants were asked to judge on each trial whether the two faces depicted the same or different individuals, allowing them to form robust representations of identity (ID) for different views of the same face. **Aversive conditioning session:** One of the two IDs used in the familiarization session in full-front view (familiar and conditioned stimulus, famCS+) was paired with a negative emotional experience (unpleasant loud sound), using a classic Pavlovian conditioning procedure with a 60% reinforcement. The other ID (familiar but not conditioned, famCS-) plus a series of filler faces (all unfamiliar) were paired with a neutral sound (soft beep noise). Importantly, all faces were always presented in front view only. Participants were instructed to look carefully at the faces and memorize them. Filler faces were added to increase encoding load and credibility of the memory task. **Main repetition priming experiment:** This session tested for any repetition priming effect when the same face ID (famCS+, famCS−, and new unknown faces) was presented in pairs of images, across different viewpoints. The famCS+, famCS-, and new unfamiliar faces (new) were presented in a rapid succession of two different images. The first image always depicted a full-front view (like during aversive conditioning), allowing for the generation of perceptual predictions, while the second image always depicted a ¾-view (either left or right deviated), and the identity of these two faces was either the same or different (repeated or non-repeated ID) in order to assess cross-view priming. Participants had to indicate whether the second face looked toward the left or right side. N.B. Faces used in the figure are home-made avatars created using FACsGen software for illustrative purpose only. Karolinska Face dataset 66 was used in the experiment.

In the current study, we investigated whether emotional learning would alter the cortical representation of faces by linking different views of the same face ID through changes in perceptual predictions. We hypothesized that the representation of an individual face in front-view may not only be strengthened after being paired with an emotional experience, but also “enlarged” to become associated with another (e.g. three-quarter) view of the same face (see conceptual framework in Fig. 1A). We therefore predicted that such changes in visual cortex would lead to the emergence of face ID priming across different viewpoints for emotionally significant, but not non-emotionally relevant faces. Such effects would demonstrate emotion-driven plasticity in cortical representations and highlight the role of experience in the development of view-invariant recognition abilities. More generally, emotion-driven plasticity in cortical representations would provide novel support to the predictive coding framework of perception by showing that affective experience may lead to novel neuronal associations in visual areas that help predict future sensory inputs, in keeping with a key role of emotion in adaptive behaviors^17^.

## Results

To probe for changes in stimulus representation in visual cortex after emotional learning, we combined a classic aversive conditioning protocol^18,19^ and a repetition-priming paradigm based on fMRI adaptation^20,21^ where different faces (conditioned and non-conditioned) were presented across different viewpoints. Repetition priming has extensively been used to investigate both memory and perception^22^. In the perceptual domain the degree to which priming generalizes across changes in certain properties of a stimulus (e.g., size or viewing angle) can be used to infer the nature of the underlying representation of this stimulus^23,24^. At the brain level, abundant work showed that repeating the same stimulus in two different formats will produce a reduced fMRI response (compared to two different stimuli) when these two different formats are encoded by the same neuronal population, but not when each format activates a separate population (i.e. “fMRI adaptation” effect)^25,26^. Moreover, repetition tasks with short lags between repeated stimuli are considered more sensitive to perceptual processing stages, as opposed to long lags more sensitive to memory effects^27^. Based on this approach, our experimental procedure was designed to probe for the response selectivity of neuronal populations representing a specific face identity within extrastriate visual cortex, and to test for any change in such representations (e.g. view specificity vs invariance) as a function of prior emotional (aversive) experience.

**Figure 2:**
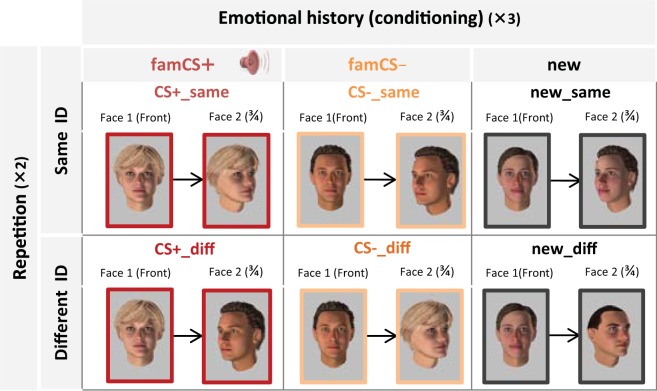
**Factorial design of the repetition priming paradigm.** The paradigm crossed 3 levels of emotional history – i.e. (1) familiar and conditioned (fam**CS**+), (2) familiar but not conditioned (famCS), and (3) novel faces (new) – and 2 levels of ID repetition (1) same and (2) different face, yielding 6 possible conditions (CS+ same; CS+ diff; CS− same; CS− diff; new_same; new_diff). *N*.*B*. *Faces used in the figure are home*-*made avatars created using FACsGen software for illustrative purpose only*. Karolinska Face dataset^66^ *was used in the experiment*.

Participants underwent fMRI scanning during three successive experimental sessions (Fig. 1B and see Materials and Methods). The ***familiarization*** **session** (Fig. 1B top) allowed them to form robust representations of two face identities (ID) linking different viewpoints from the same person (“**fam**” faces). In the subsequent ***conditioning*** **session** (Fig. 1B middle), an aversive emotional value (unpleasant loud sound) was paired with one of the two IDs used in the familiarization session (conditioned stimulus, **CS**+) (i.e. emotional learning phase). The other face ID (**CS**−) was paired with a soft, neutral sound (pseudo-random assignment across participants). Critically, the third phase was the main experimental session during which participants performed a ***repetition priming*** **task** to test for the emergence of a shared cortical representation for the two viewpoints of the emotional identity (**CS**+), but not for the neutral identity (CS−). The repetition priming task exploited a “paired-stimulus adaptation” design in which a pair of either the same or different images are presented in rapid succession^20,21,28,29^, a procedure eliciting robust decreases in fMRI responses in brain areas that are sensitive to the repeated properties of stimuli in a pair, and probing perceptual rather than mnemonic stages of priming^25,27^. By using this paradigm, we could therefore test for differential repetition priming effects when the same face ID was presented across different viewpoints in pairs of images, as a function of previous emotional experience with this face (Fig. 1B bottom). Overall, the repetition priming phase constituted a 3 × 2 design with 3 levels of emotional history [**famCS**+ (i.e. familiar, conditioned face), **famCS**- (i.e. familiar, unconditioned face), and **new** (unknown face)], as well as 2 levels of ID repetition (same, different), yielding 6 face pairs/trial types in total (**famCS** + **same**; **famCS** + **diff**; **famCS− same**; **famCS− diff**; **new same**; **new diff**) (Fig. 2).

Our main analyses focused on the repetition priming experiment in order to probe for differential repetition priming across viewpoints as a function of prior emotional (aversive) experience. Specifically, we hypothesized that prior emotional experience may create new perceptual predictions in response to the emotional face ID (fam**CS**+) and thus activate the cortical representation of the other viewpoint of the same face, leading to greater repetition priming effect for pairs containing the fam**CS**+ face during the repetition priming task. Critically, this other viewpoint never appeared during the conditioning phase itself. In contrast, we expected no priming across viewpoints for the famCS- or for new faces, in accordance with previous evidence for view-specific repetition effects during face recognition^30,31^. New faces were also used as a control condition to distinguish effects of emotional learning from non-specific repetition priming due to familiarity^27^.

The effectiveness of our conditioning procedure was verified in several ways. First, brain imaging results during the emotional learning phase revealed a widespread activation of visual and auditory areas together with amygdala, insula, and other subcortical areas implicated in emotion processing (see suppl. Materials and Fig. S1, Table S1). This accords with previous studies on aversive learning^18,19,29^, and shows robust recruitment of limbic areas mediating aversive learning, together with sensory areas encoding the CS and US information. In addition, during the subsequent repetition priming phase, both fMRI and behavioral responses indicated successful acquisition of aversive value for the famCS + faces (relative to famCS- faces), persisting beyond the conditioning session (see Suppl. material and below).

### Main emotion effects: differential brain responses to faces after aversive learning

**Figure 3:**
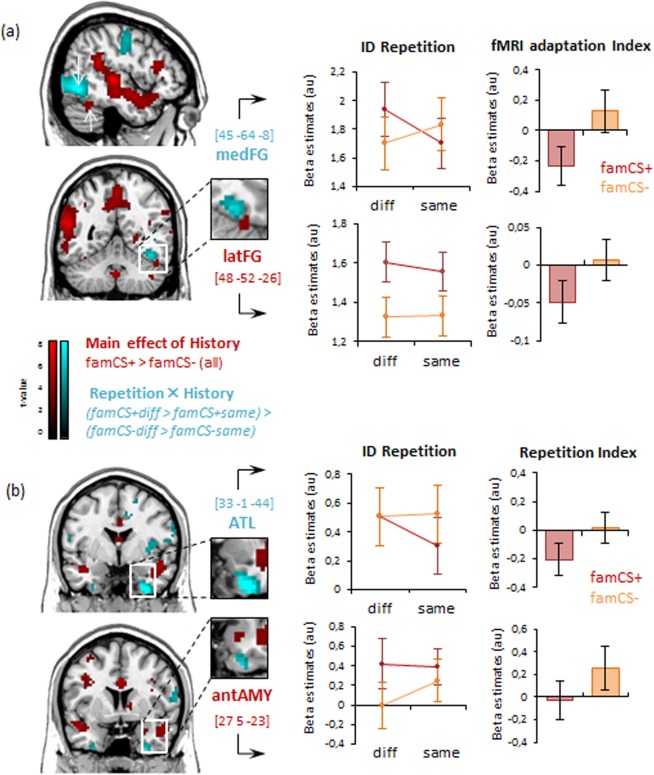
**Brain areas showing emotional history-related increases (in red) and repetition-related decreases (in blue) as a function of emotional learning in the priming session** (see also Tables 1, 2). **(a**) Whole brain maps indicate a functional-anatomical segregation within the FG with the latFG showing a main effect of emotional history and the medFG showing a selective repetition suppression effect for famCS+ faces. **(b**) Whole brain maps show history-related increases in anterior amygdala (AMY), and a history x repetition interaction in the anterior temporal lobe (ATL). For both a) and b) plots of the activity parameters illustrates beta weights as a function of face ID repetition. An index of cross-view repetition suppression was calculated by subtracting parameters to same view condition from the different view condition. Note that plots here are shown to illustrate the results but no post-hoc statistics were performed on these data.

Whole-brain statistical maps of brain activity during the repetition priming experiment were computed using a random-effect flexible factorial model, crossing the two factors of EMOTIONAL HISTORY (i.e. conditioning) and FACE ID REPETITION (see Materials and Methods). We first confirmed that emotional learning successfully took place by comparing brain responses to faces associated with different past experiences (aversive vs neutral association). To this aim, we examined the main effect of face history, regardless of ID repetition, by contrasting all trials with the fear-conditioned image to those with the non-conditioned image, i.e., *famCS* + (*same* + *diff*) > *famCS−*(*same* + *diff*). Greater responses were found in several areas of the face processing network (Table 1), including bilateral lateral fusiform gyrus (FG) (Fig. 3a, red), right anterior amygdala (Fig. 3b, red), and extensive cortical regions within and around the superior temporal sulcus (STS) (Fig. 3a, red). These increases indicate enhanced perceptual and social-affective analysis of faces after they have been associated with aversive value through past emotional experience, irrespective of ID repetition across the successive viewpoints. Such boosting accords with generally heightened attention and deeper perceptual processing for emotionally salient stimuli^1^, but unrelated to perceptual predictions or associations across changes in sensory inputs.

**Table 1:** Main effect of emotional history in the main repetition priming session.

Contrast	Side	Structures	MNI cords			t	Cluster Size
			x	y	z		
**Main effect of emotional history**							
famCS+ (same+diff) > famCS− (same+diff)	L.	Post STS	−57	−46	19	8.19	144*
	L.	Mid STS	−48	−19	−5	5.17	—
	L.	Temporal pole	−39	−10	−17	5.77	17*
	R.	Post STS	66	−31	7	7.55	156*
	R.	ant STS	51	−10	−11	5.84	13*
	R.	Lateral FG (FFA)	48	−52	−26	4.11	22*
	L.	Lateral FG	−36	−55	−20	3.57	5 ~
	R.	Amygdala (AMY)	27	5	−23	3.58	5 ~
	L.	dlPFC	−48	23	19	5.33	594 #
	L.	vlPFC	−30	26	−5	5.09	—
	L.	Primary motor cortex	−30	−20	31	5.03	—
	R.	dlPFC	42	23	19	5.14	160 #
	R.	midFG	42	8	31	3.38	—
	L.	postCG	−3	−16	34	5.09	15*
	R.	ACC	3	8	28		—
	L.	Precuneus	−3	−64	49	5.98	84*

Brain areas showing a history-related increases (see also Fig. 3, in red). Coordinates (MNI space) refer to maximally activated foci: x = distance (mm) to the right (+) or the left (−) of the mid sagittal line; y = distance anterior (+) or posterior (−) to the vertical plane through the anterior commissure; z = distance above (+) or below (−) the inter-commissural line. L and R refer to the left and right hemisphere, respectively. p values and their corrections are indicated by the symbols next to the voxel sizes. *p < 0.05 FWR corrected at the peak level for the whole brain (random-effect analysis), ~ p < 0.05 Small Volume Corrected (SVC), # p < 0.001 uncorrected. – indicate that the structure is part of the previously listed cluster.

### View-dependent repetition effects as a function of emotion-driven prediction

We next turned to the main question of our study, concerning whether emotional signals induced specific perceptual predictions (i.e., concerning the CS + face ID) through an alteration of visual representations within face-responsive brain regions, so as to link different views of the same face. This issue was tested by fMRI adaptation effects in our “paired stimulus” paradigm^20,21,26^, whereby two successive stimuli should elicit weaker responses when they activate the same neuronal population as compared with when they activate distinct populations. Accordingly, we predicted that an adaptation effect should occur if, and only if, the first face front-view activates a neural representation that also encodes the same face in side-view (same ID condition). In contrast, no fMRI adaptation should occur if the front-view and side-view of the same face activate different representations^25,31^, similar to image pairs depicting different persons (different ID).

We first tested for a main effect of ID repetition (always involving two views of the same face), independently of prior conditioning, by computing the contrast *allDiff* > *allSame* that included famCS + , famCS-, and new faces (whole-brain ANOVA). No significant repetition suppression was found in visual areas, but only reduced response in bilateral inferior frontal gyrus (IFG) (Table 2). This accords with previous studies of face priming^7,9^ reporting that extrastriate visual areas in FFA and OFA hold predominantly view-specific representations and show no or weak repetition suppression across different viewpoints.

**Table 2:** Main effect of repetition (top) and interaction between emotional history and repetition (bottom) in the priming session.

Contrast	Side	Structures	MNI cords			t	Cluster Size
			x	y	z		
**(a) Main effect of Repetition**							
allDiff > allSame	L.	IFG	−39	14	19	3.60	10 #
	R.	IFG	48	8	25	3.35	5 #
famCS+diff > famCS+same	R.	SFG	21	20	55	3.81	26 #
	L.	SFG	−18	17	58	3.57	20 #
	R.	Med FG	6	−22	61	3.63	13 #
	R.	Fusiform gyrus (FG)	45	−58	−14	2.86	10 ~
**(b) Emo History x Repetition interaction**							
(famCS+diff>famCS+same) > (famCS-diff>famCS-same)]	R.	Dorso-medial FG	45	−64	−8	7.39	231*
			42	−58	−17	7.18	—
	R.	IOG (OFA)	45	−82	−2	6.37	—
	L.	Dorso-medial FG	−42	−76	−5	5.25	390 #
	L.	IOG (OFA)	−21	−97	25	5.87	—
	R.	ATL/Parahippocampal	33	−1	−44	7.04	180 #
	R.	ATL/entorhinal	18	−16	−26	4.91	—
	L.	ATL/entorhinal	−24	−10	−26	5.36	52 #
	L.	ATL/ Parahippocampal	−30	−10	−35	3.57	11 #
	R.	Med. Frontal G.	15	−13	73	6.12	125*
			9	−16	64	5.69	—
	R.	Insula	36	−4	−4	4.74	70 #

Brain areas showing a repetition-related decreases (see also Fig. 3, interaction in blue). Same conventions for coordinates as Table 1. *p < 0.05 FWR corrected at the peak level for the whole brain (random-effect analysis), ~ p < 0.05 Small Volume Corrected (SVC), # p < 0.001 uncorrected. – indicate that the structure is part of the previously listed cluster.

Strikingly, however, when the same analysis was performed on the critical trials with aversively conditioned faces only (*famCS* + *diff* > *famCS* + *same*), we now observed a reliable repetition effect in the right FG [45 -58 -14], reflecting view-independent responses to the same face ID (Table 2). This indicates that seeing the CS + face could activate a representation of the same face in a different viewpoint, leading to selective fMRI adaptation to stimulus pairs in this condition. In contrast, no such effect was found in FG or any other visual area when analyzing only the previously familiar, but unconditioned faces (*famCS*-*diff* > *famCS*-*same*) or the new faces (*newDiff* > *newSame*), indicating no adaptation across viewpoints for this face. Therefore, the unique repetition-priming effect for CS + faces cannot be explained by previous exposure to faces and their different viewpoints, since CS + and CS- stimuli were equally often seen during the familiarization and conditioning phases (and counterbalanced across participants).

Critically, we formally confirmed this difference in view-independent repetition priming as a function of emotional history (reflecting emotion-driven perceptual predictions) by directly computing a REPETITION × EMOTIONAL HISTORY interaction contrast [(*famCS* + *diff* > *famCS* + *same*) > (*famCS*-*diff* > *famCS*-*same*)], using the same whole-brain ANOVA as above (Table 2). This revealed significantly greater repetition effects for famCS + than famCS- face ID in occipito-temporal visual cortex including the dorso-medial part of the FG and the inferior occipital gyrus (IOG) (Fig. 3a, blue). Interestingly, a similar effect was also observed in more anterior temporal lobe (ATL) areas bilaterally around the entorhinal and parahippocampal cortex, with a larger extent in the right hemisphere (Fig. 3b, blue). No significant activation was found for the opposite interaction contrast (repetition effects for famCS- > fam**CS**+).

Overall, these results reveal repetition priming effects that generalized from front to three-quarter views only for conditioned faces within face-responsive brain regions, reflecting a view-independent encoding of ID information that is selective for emotionally significant stimuli. Note that three-quarter views were *never* presented during the conditioning phase itself. Hence, priming effects from front to three-quarter views can only be explained by changes within cortical areas representing each viewpoint that allowed linking one view to the other after emotional learning; whereas these two views remained distinct and did not prime from one to another for faces with a neutral emotional history.

Moreover, it is notable that repetition priming effects appeared to selectively recruit the dorsomedial part of the FG, rather than the adjacent lateral part usually associated with the FFA proper, as well as a distinct, more anterior area in medial/ventral ATL. These data suggest that these regions may hold ID-specific and/or view-invariant memory traces of faces (i.e. abstract 3D representation), unlike the FFA proper.

We also note that these repetition effects were not due to face familiarity alone, but specifically induced by the prior emotional history of faces, since both the famCS + and famCS- faces were seen equally often (in all experimental phases). These effects were also distinct from non-specific repetition priming due to prior exposure, as defined by comparing trials with new faces relative to all familiar faces (New faces > famCS + and famCS-) which highlighted widespread increases in ventral extrastiate visual areas in accordance with prior studies^27^.

### Functional Segregation for Face ID Representation within Face-Selective Brain Regions

To further characterize the functional organization of brain areas subserving face ID representation, we compared activation patterns described above with those obtained in the same participants during an independent “face localizer” scan (see Materials and Method). Contrasting blocks of faces versus non-faces from this scan revealed strong activations in the temporal and occipital lobes of both hemispheres, including bilateral fusiform regions corresponding to the FFA, bilateral occipital regions corresponding to the “occipital face area” (OFA), plus bilateral posterior STS and amygdala (Fig. S2a, Table S2). These face-responsive regions were then used to further analyze results from the main repetition priming experiment.

Inspection of activation peaks revealed that the face-selective FFA (in yellow) overlapped mostly with the lateral FG portion showing increase activity related to past conditioning history (i.e., emotional boosting, Fig. S2a, top), as consistently observed in other studies^1^. Further, inspection of activation parameters across conditions (betas) for the FFA cluster, as defined by the localizer showed clear emotion and familiarity effects, but no modulation by ID repetition (Fig. S2b). When submitting these parameters to ANOVAs outside SPM (ROI analyses), we found a main effect of emotional history (F = (2,42) = 9.16, p = 0.00; famCS + > famCS-: p = 0.01) and a main effect of hemisphere (Right > Left, F(1,21) = 11.52, p = 0.002), but no view-independent priming effects (p = 0.92) and no interaction (Fig. S2b). These results demonstrate that the lateral fusiform cortex corresponding to the FFA proper may not contain an abstract 3D-representation of face identity generalizing across viewpoints, but rather encodes image-based (i.e. view-dependent) information that is boosted by its emotional relevance. In contrast, the more dorsomedial fusiform cortex, just adjacent to the FFA proper (Fig. S2a), shows a robust view-independent repetition-related decrease in activity that is selective for CS + faces (Fig. 3a, medFG in blue), when their three-quarter view is preceded by front view, in keeping with our hypothesis of emotion-driven perceptual predictions that allow for linking different traces of the same stimulus within the visual cortex (i.e. view-independent representation).

On the other hand, inspection of occipital activations revealed that the face-selective OFA defined by the face localizer scan did overlap with the IOG cluster showing an ID repetition x history interaction in our SPM analysis above (Fig. S2a, top in green). A separate ANOVA on activity parameters from the OFA ROI found the same interaction on the right side (F(2,42) = 5.59, p = 0.007), reflecting a significant priming-related decrease for the famCS + condition (p = 0.023) as opposed to a priming-related increase for the famCS- (p = 0.028) (Fig. S2a,b). This indicates that, unlike FFA proper, the functionally defined OFA may hold both view-independent and face-selective information.

Finally, activations in medial temporal lobe highlighted a partial overlap (green, Fig. S2, bottom) between the face-selective responses in amygdala (in yellow) and regions showing differential repetition-related decreases as a function of emotional history (cyan, Fig. S2, and bottom). Only the most anterior sector of ATL corresponding to the rostral parahippocampal cortex (PHC) showed repetition-related effects without face selectivity (cyan only). An ANOVA of the face-selective amygdala ROI showed a main effect of emotional history (F(2,42) = 5.42,p = 0.007) and a repetition x history interaction (F(2,42) = 3.26, p = 0.04) (Fig. S2b). Post-hoc analyses indicated higher responses to famCS + than famCS- trials (p = 0.024) but lower activity to the famCS + repetition than famCS- repetition (p = 0.037).

## Discussion

Our results provide new insights concerning the influence of emotional learning on perception and neural plasticity in sensory cortices, but also extend our knowledge on the substrates of view-invariant face representations and their remodeling by affective signals. First and foremost, we show that following emotional experience, the perception of a threat-related stimulus activates the representation of another image of the same stimulus and thus leads to robust repetition priming across different views. Such effect was not observed for an equally familiar but non-conditioned stimulus. This remarkable pattern suggests that emotional learning can remodel the cortical representation of a visual stimulus and link different images of the same face identity that otherwise are separately coded. These findings fully accord with our prediction of emotion-driven plasticity in the cortex (see introduction). Such linkage between different stimulus representations could generate perceptual predictions that help encode new sensory inputs when these are distinct from, but strongly associated with, emotionally significant cues encountered during the initial aversive experience. Second, our results reveal a remarkable functional-anatomical segregation within the face processing network with selective coding of view-invariant ID information in the medial part of the fusiform cortex, as well as in more anterior temporal lobe (ATL in parahippocampal and enthorinal areas). In contrast, lateral sectors of the fusiform cortex were found to held view-specific representations of face ID, and did not show repetition priming effects across viewpoints even after emotional learning. Taken together, these findings add to previous work suggesting distributed representations of familiar faces in the brain and provide new insights on view-invariant processing in the human visual cortex.

### Boosting of perceptual face processing and learning in the FFA and amygdala

We observed conditioning-related increases in FFA and AMY responses to faces, indicating enhanced perceptual analysis and affective appraisal of behaviorally-relevant stimuli. FFA increases were independent of ID repetition across successive images and likely to arise from feedback modulatory signals from the amygdala^1,32^, since visual features and familiarity did not differ from other conditions. It is usually assumed that such modulation of sensory processing by emotion allows for rapid attention toward potential threats and thus promotes rapid behavioral responses^1,33^. AMY activation increases are consistent with evidence that the amygdala contains specific experience-based (not instruction-based) memories that subserve emotional learning^34^, and agree with a role of the amygdala in the tuning of fusiform responses to emotionally significant faces.

Feedback signals from the amygdala onto visual areas might also contribute to forming new or more robust representations of emotional stimuli in the cortex itself. This idea is consistent with animal studies showing that pairing a stimulus with an aversive experience can shift the tuning curves of sensory neurons towards the characteristic features of the conditioned stimulus and/or increase the number of neurons representing these features^35,36^. Damage to the amygdala may abolish these changes^37^, pointing to a role for amygdala feedback in driving cortical plasticity in addition to more immediate modulation of attention^38^. Further, our results suggest that such emotion-driven plasticity might increase connections between neurons representing different features of the same CS + stimulus, and thus contribute to activate larger neuronal populations in response to emotionally significant cues. Specifically, in our study, neurons selective to a particular face viewpoint might become more effectively linked to neurons responsive to other viewpoints of the same ID, leading to their co-activation during the presentation of only one of the viewpoint. Alternatively, the same neurons might become more responsive to other features or other views of the same stimulus. In both cases, this would lead to repetition priming effects across views, as observed here.

More broadly, this mechanism would accord with predictive coding models that consider perception as an inference process of matching (learned) top-down predictions against bottom-up sensory evidence. Predictive coding posits that the neural response of category-selective cortices reflects a dynamic reciprocal interaction of activity related to prediction (i.e., “perceptual priors” based on previous experience) and prediction error (i.e. “perceptual surprise” generated by current input), rather than a serial process of feature detection and integration^39,40^. Our results provide novel evidence in favor of this framework by showing that visual cortex activity varies as a function of the past history and affective meaning of a particular face, such that seeing a fear-conditioned face viewpoint may recruit connected representations that will reduce “perceptual surprise” in the prediction error process and thus lead to repetition priming effects on trials with different viewpoint of the same face ID. Note that in our paradigm, three-quarter views were never presented during the fear learning phase, such that priming by a front-view of the famCS + face (but not famCS-) could only occur through emotion-induced processes that linked these two views within the cortex and could thus implicitly predict the three-quarter viewpoint upon seeing the front viewpoint (an effect that did not occur for famCS- faces).

Importantly, here we show that perceptual predictions may powerfully be shaped by emotional learning processes. On the one hand, this points to a major role for emotional signals in guiding neural plasticity in perceptual pathways, possibly by strengthening associations between different representations of the same stimulus in the cortex. Such plasticity would constitute a highly adaptive mechanisms enabling more efficient detection of emotionally-relevant stimuli in response to varying sensory cues in naturalistic environment where stimuli may not repeat in the exact same conditions. On the other hand, these data converge with a recent hypothesis proposing that face recognition might operate through matching processes that compare top-down predictions built in higher-level cortical areas with bottom-up sensory inputs, such that representations stored in the higher-level areas can influence neural responses in the lower-level areas^41^.

We note the current effects differ from fear generalization^3,42^, where emotional responses to a given stimulus spread to other stimuli that are perceptually similar (e.g., nearby hues or shapes when the CS + is a colored disk) or semantically similar (e.g., other animals when the CS + is a dog). Here, we did not test whether similar aversive responses were evoked by another viewpoint of the CS + face, another resembling face ID, or any other face in general relative to a different object category. Instead, we demonstrate that two previously distinct representations of a specific visual stimulus (encoded by distinct neuronal pools) become integrated following emotional learning (now subserved by partly common neuronal pools), so that activating one representation will prime the other representation after conditioning (but not prior to it).

In addition, in our study, we also found increased activation to the fear-conditioned face in large parts of the STS, as well as in PCC, ACC, insula, and PFC, all presumably downstream to face recognition in visual cortex. This accords with a general involvement of these regions in higher stages of face processing including multisensory integration and social appraisals^43,44^, retrieval of contextual memory^45^, as well as cognitive control related to decision making and action^46,47^. Furthermore, several regions with the dlPFC and vlPFC were recently proposed to mediate emotional prediction processes in the service of adaptive behavior^48^, and these regions also activated in our study in response to faces associated with prior aversive events (e.g. see Table 1). Taken together, these data accord with our predictive coding perspective on emotion processing and learning. Specifically, we propose that novel sensory predictions may be formed subsequent to affective appraisal for salient visual stimuli and then utilized during subsequent encounter with different views of the same stimuli, possibly implicating feedback interactions between higher and lower processing levels within the visual system hierarchy.

### View-invariant Identity Coding in the medial FG, OFA, and ATL

Another novel finding of our study is that repetition priming effect generalizing across views for CS + faces did not occur in the FFA proper (in lateral fusiform) as identified by a standard localizer, but rather in an adjacent, more medial, portion of the fusiform. In itself this result provides new insights concerning the functional organization of face recognition in the human visual cortex. Previous studies reported no overall preferential responses to front-views or 3/4-views in the FFA^49,50^, but view-dependent repetition effects occurring only when faces are repeated with the same view (either front or 3/4), not across different views^9,10,30,31^. Taken together, these results suggest that the representation of face ID in the FFA may primarily operate on view-dependent information^51,52^, without building a full 3D representation. The integration or linkage of multiple views of the same face might be computed through interpolation mechanisms^13,14^ which could depend at least partly on fusiform regions adjacent to the FFA proper. In any case, our results converge with others^7^ demonstrating that, unlike the FFA in lateral fusiform, the medial FG may exhibit repetition priming effects across different views of unfamiliar faces and therefore hold more abstract 3D representations of face ID. The medial FG was also proposed to mediate multi-featural encoding mechanisms that link different face parts together^53^.

Remarkably, view invariance in repetition effects for the fear-conditioned face ID was also found in the OFA and ATL. This suggests that aversive conditioning may promote a remodeling of face representations subserving identity-specific codes in distributed cortical areas of the ventral visual stream. The ATL is engaged in social as well as semantic and episodic memory^54,55^. A role in face processing and ID recognition was also recently emphasized^11,56,57^, perhaps implementing a key node at the interface between the extraction of visual face cues and access to memory information about people^7,58,59^. This area may underpin the abstract “person identity nodes” postulated by classic cognitive models of face recognition^60^, distinct from the visual “face recognition units” presumably formed in extrastriate cortex. Studies in monkeys accord with this proposal by showing clear identity-selective and view-invariant tuning in the most anterior face patch (AM) within the temporal lobe^61^—which may be the homologue of the face-responsive area in ATL^62^. On the other hand, the OFA was also responsive to different views of emotionally significant faces, pointing to an involvement of this visual area in higher-level mechanisms of face recognition^63^, rather than just low-level processes^64^. This accords with the notion that high-level prediction error signals may propagate to low-level areas in the macaque face processing hierarchy (ML patch) and thus tune their responsiveness according to representations stored in higher-order face patches (AL/AM)^41^.

In sum, our novel results fit our predictions based on predictive coding model of perception and go beyond previous work on emotional guidance of perception, attention, and memory by demonstrating that prior emotional experiences may lead to changes in response selectivity of high-level visual cortex. Whether these cortical changes are transient or long-lasting, and whether there are specific to threat signals or may extend to positive emotional experiences or rewards, remains to be determined. Such changes may serve to generate perceptual predictions in response to emotionally significant information, guiding and facilitating recognition of behaviorally relevant stimuli across varying sensory inputs. This may occur through remodeling or connecting different neuronal representations of the same stimulus. Further, in the human face recognition system, such effects might differentially recruit the medial rather than lateral fusiform areas, as well as the inferior occipital cortex and anterior temporal lobe, unveiling a functionally segregated but anatomically distributed representation of face identity. These results shed new lights on neural mechanisms by which affective signals can govern perceptual processes and drive neural plasticity.

## Materials and Methods

### Population

We recruited 27 healthy volunteers (11 females, mean age ± s.d. = 24.3 y ± 3.5 y) from the general student population at the University of Geneva. Based on our previous work^53^, this sample size was expected to have sufficient power for unveiling emotion-driven modulations in visual cortex, with a probability of replicating such effects of >90% for right FFA and >99% for right OFA^65^. A semi-structured interview established the absence of neurological, psychiatric disorders, or drug use. Five participants (2 females) were excluded from fMRI analyses due to movement artefacts during the scanning session or lack of aversive conditioning (see supplementary information). Each participant had normal or corrected-to-normal vision. Thus, in total, 22 subjects were included. This study was approved by the Neurosciences Cliniques Ethics Committee of the University Hospital of Geneva. All participants provided written informed consent according to local ethics guidelines and received a financial compensation for their participation.

### Experimental design

#### STIMULI

Stimuli were digitized color images (562 × 762 pixels) of 64 different face identities (32 males and 32 females) with neutral expressions, taken from the Karolinska Face dataset^66^. Face stimuli had two different head orientations, either full-front or 3/4-rotated with the head towards the left or right. Gaze direction was always congruent with head direction. This resulted in a total stimulus set of 192 images, i.e., 64 identities (IDs) by 3 views. In addition, scrambled low-pass versions of these pictures were generated using Matlab (R2009b, MathWorks Inc., Sherbom,MA) scripts and served as visual masks. All these images were used in three successive experimental phases described below (i.e. *Familiarization*, *Conditioning*, and *Repetition Priming*) performed by the participants in a single session (Fig. 1B).

#### PARADIGM

Participants underwent fMRI scanning during three successive experimental sessions: face familiarization, aversive conditioning, and repetition priming (Figure 1abc). In all tasks, visual stimuli were presented using the Cogent2000 toolbox for Matlab (MathWorks Inc., Natick, MA) on a back-projection screen inside the scanner bore using an LCD projector (CP-SX1350, Hitachi, Japan). Faces were displayed on a light gray background, with a visual angle of 6.6° vertically and 4.7° horizontally. Responses were made on buttons (assigned to Yes/No, and Left/Right labels) using an MRI compatible serial response box (HH-1 × 4-CR, Current Designs Inc., USA).

Face familiarization. The *familiarization session* (~4mn) allowed participants to learn individual faces across different views (Fig. 1B top). For each participant, two IDs (1 male and 1 female) were randomly selected in order to be used as familiar faces in the next experimental sessions. Each trial started with a central fixation cross (mean duration = 2.75 s; range 2 to 3.5 s). Then, a pair of faces in front and 3/4 views were presented (500 ms each, visual angle 5.4° × 5.4°) in rapid succession, always separated by a scrambled image (250 ms) to limit retinal persistence and overlap between the two faces. At the end of the trial, participants gave their response on a keypad (‘same/different’; maximum response time: 2 s). In total, 30 pairs of faces were generated with the 2 selected IDs (#ID1 and #ID2) presented in either (a) full front, (b) 3/4 left, or (c) 3/4 right viewpoint (15 combinations of two images from the same face, presented in two possible order). The first picture was a full front or a 3/4 view (left or right) for half of the trials each.

Aversive conditioning. The *conditioning session* aimed at randomly associating an aversive emotional value with one of the two face IDs used in the familiarization session (conditioned stimulus, famCS+), while the other face ID was seen equally often but remained emotionally neutral (famCS−). Conditioning was achieved by pairing a given face picture (one familiar ID out of the two, in full front viewpoint) with an unpleasant loud sound, composed of 3 bursts of white noise (125 ms each, separated by 50 ms silent gaps) (Fig. 1B middle). The other face was paired with a soft, neutral sound. The session (~7 mn) comprised 30 presentations of the *famCS*+ face ID and 30 presentations of the *famCS*− face ID, mixed with 10 new filler faces, all in randomized order (70 trials in total). Importantly, the conditioned face ID was now always presented in front view. Each trial consisted in a fixation cross (mean duration = 2 s; range 1.5 to 3 s), followed by a face stimulus (3 s) associated with either the aversive (fam**CS**+) or the neutral sound (famCS- and filler faces). A few longer inter-trial intervals were introduced after a series of 10 faces (5 s fixation cross) to prevent visual fatigue and limit saturation in the fMRI BOLD signal. Similar to classic partial reinforcement procedures (e.g., Sehlmeyer *et al*.^42^), the aversive sound co-terminated 60% of the famCS+ presentations, with the remaining 40% associated with the same neutral sound as the CS− face. This procedure allowed a consistent pairing between the selected face ID and the aversive sound, without habituation, and ensured that conditioning effects were driven by sound aversiveness rather than just multimodal associations. Importantly, only the full front view was conditioned, no 3/4 views were presented in this session. This was because previous work showing that it was more difficult to generalize from the front viewpoint to three-quarter than vice versa^30^, hence making our predictions more stringent and our results more robust.

The white noise volume of the CS+ event was adjusted for each individual at a very unpleasant yet not harmful level during a preliminary calibration phase, prior to scanning. More precisely, the subjects were presented with a white aversive sound at a volume of 70 dB using headphone while passively lying in the MRI. They were asked to indicate their maximal hearing threshold by increasing or decreasing the volume of this sound using left or right button presses. Depending of the participants, selected subjective volume ranged between 62.5 and 34 dB (mean ± s.d = 49.03 ± 7.06 dB). Crucially, to ensure that the subject were not too “self-protective” and that our stimuli were indeed aversive, we then manually increased this maximal subjective threshold by extra 2.5 dB and set this value as default volume across the next experimental scanning sessions.

Main repetition priming experiment. This third session constituted the main experimental phase and tested for any repetition priming effect when the same face ID (fam**CS**+, famCS−, and new unknown faces) was presented in pairs of images, across different viewpoints. We used a similar design as during the familiarization session (Fig. 1B bottom) except that, at the end of the trial, the participants now indicated whether the second face looked to the left or right side by using a response keypad (‘left/right’; within 2 s). Importantly, in this session, the first picture (famCS + , famCS-, or New) always depicted full-front faces (i.e., the viewpoint used during aversive conditioning), while the second picture always showed 3/4 views (left or right) whose identity could be either the same or different from the first face (i.e., repeated ID or not). To minimize any possible extinction of aversive conditioning through this session due to the repeated presentation of the famCS + , the aversive loud sound was delivered in 10% of the famCS + trials (2 among 20, trials removed from the fMRI analyses of conditioning effects). Twenty trials per condition (120 in total) were presented using an event-related design during approximatively 15mn. This repetition priming phase constituted a 3 × 2 design with 3 levels of emotional conditioning (fam**CS**+, famCS-, News) and 2 levels of ID repetition (same, different), yielding 6 face pairs/trial types (famCS + same; famCS + diff; famCS− same; famCS− diff; new same; new diff) (Fig. 2).

Face localizer session. Additionally, to identify face-responsive regions in individual brains, subjects performed a 1-back task during blocks of neutral faces, fearful faces (NimStim Face Set, Tottenham *et al*., 2009), houses and scrambled ovals (4 conditions, 80 pictures per condition). Face localizer stimuli were identicial as those used in a previous study (Meaux & Vuilleumier, 2016), pictures of houses were stimuli used in Vuilleumier, Armony, Driver & Dolan (2001), and scrambled were generated from faces using matlab scripts. All pictures were gray-scale photographs. We scanned 8 sessions of 4 blocks, each block containing 10 pictures of each type, presented for 500 ms with an ISI of 50 ms. Subjects had to press a button whenever a stimulus was presented twice in a row. The inter-block interval was 3 s and blocks were presented in random order.

### Imaging Protocol and Statistical Analysis

#### Mri data acquisition and preprocessing

MRI images were acquired during the three experimental sessions using a 3 T whole-body MRI scanner (Trio TIM, Siemens, Germany) with a 32 channel head-coil. Functional images were acquired using a multiplexed EPI sequence (Feinberg *et al*., 2010) with TR = 650 ms, TE = 30 ms, flip angle = 50°, 36 slices, 64 × 64 pixel, 3 × 3 × 3 mm3 voxel size, and 3.9 mm slice spacing. The multiband acceleration factor was 4, and parallel acquisition technique (PAT) was not used. Structural images were acquired with a T1 weighted 3D sequence (MPRAGE, TR/TI/TE = 1900/900/2.27 ms, flip angle = 9 degrees, PAT factor = 2, voxel dimensions: 1 mm isotropic, 256 × 256 × 192 voxel).

Functional images processing and analyses were carried out using SPM8 (Wellcome Department of Cognitive Neurology, London, UK) (http://www.fil.ion.ucl.ac.uk/spm/). The first 10 volumes were excluded from analysis to account for T1 saturation effects. Functional images were realigned to the mean image of each session by rigid body transformation, coregistered with each participant’s structural image, spatially normalized to the standard Montreal Neurological Institute (MNI) EPI template, resampled to an isotropic voxel size of 3 mm, and finally smoothed with an isotropic 8 mm full-width at half-maximum Gaussian-kernel.

To ensure that we do not have any major startle (i.e. motion spikes) across our participants, we checked that the motion parameters were never above a threshold of 3 mm for translation parameters (x, y, z), and 3 degrees for rotation parameters (yaw, pitch, roll) throughout the entire time-series of both the conditioning and main repetition priming sessions, whatever the conditioning (i.e. emotional history) condition was. Furthermore, the 10% CS + trials associated with the aversive sound where removed from the analyses of the main repetition priming experiment.

#### Mri statistical analyses

Critically, our goal was to determine whether face ID emotional relevance acquired throughout the conditioning session (see supplementary information) triggered subsequent modulation of it representation within sensory cortices. Abundant work has shown that repeating a given stimulus in two different formats (e.g. views) will produce a reduced fMRI response (compared to two different stimuli) when these two different formats are encoded by the same neuronal population, but not when each format activates a separate population^24–26,67–69^. Accordingly, to reach our aim, our analyses focused on the analysis of brain activity during the main repetition priming experiment.

Functional MRI data acquired in the repetition priming sessions were analyzed using the general linear model (GLM) implemented in SPM8. For all analyses, activations were considered as significant when exceeding a threshold of p < 0.05 FWE corrected for multiple comparisons across the whole brain, with an underlying voxel height threshold at p < 0.001 uncorrected (t(168) >3.14).

Main repetition priming experiment. For individual analysis (first level), trials onsets from the 6 conditions of interest (Fig. 2) (i.e. CS + same; CS + diff; CS− same; CS− diff; new_same; new_diff) were modeled in the design matrix as separate regressors, convolved with the canonical HRF function. To account for movement-related variance, the first-level GLM also included realignments parameters from each session [x, y, and z translations and pitch, roll, and yaw rotations] and their temporal derivatives as covariates of no interest. Low-frequency signal drifts were filtered using a cutoff period of 128 s. After model estimation, contrast images were calculated for each experimental condition (vs baseline). The resulting individual maps of t-statistics were then used for the group (second-level) random-effect analyses. Whole-brain statistical maps were computed using a flexible factorial design^70^, under the assumption of unequal variance between subjects. Contrast images probing for the long-lasting effect of aversive CONDITIONING, regardless of face repetition, were computed by comparing *[famCS* + (*same* + *diff*)) vs (*famCS−* (*same* + *diff*)]. Brain regions sensitive to face REPETITION were determined by comparing *all*_*Diff* vs *all_Same* ID pairs (testing for view-independent repetition effects, regardless of conditioning). Finally, the CONDITIONING × REPETITION interaction was determined by comparing *[famCS* + (*diff*-*same*)] × *[famCS*- (*diff*-*same*)] (highlighting distinctive adaptation for conditioned versus non-conditioned face IDs).

Face Functional Localizer and ROI analysis. In addition to the statistical analysis performed across the whole brain for the different conditions of interest, results from the main repetition priming experiment were also examined within face-selective regions that were independently defined by a preliminary face localizer scan. Standard analyses using the GLM in SPM8 were performed in a manner similar to the approach described above. Contrast images reflecting the differential activity to Faces (fearful and neutral) vs Non-Faces (houses and ovals) were calculated for each participant. These contrasts were then fed in a second-level repeated-measure ANOVAs (flexible factorial design), under the assumption of unequal variance between subjects. Group activation peaks were identified in the bilateral pSTS, FFA, OFA, and amygdala (AMY) (see suppl. Table 2) and were reliably identified across participants. ROI analyses were performed with group level clusters. Brain regions showing face-selective responses in the localizer scan were delimited as a mask (inclusive) and then used to test for conditioning and repetition effects in the experimental scans. Activity parameters (β estimates) from each condition of interest (famCS+ same; famCS+ diff; famCS− same; famCS− diff; news_same; news_diff) for each participant and each ROI were extracted and submitted to repeated-measure ANOVAs and post-hoc comparisons. ROI analyses were conducted with the SPM extension of MarsBaR toolbox^71^.

## Supplementary information

Efficiency of the conditioning session
