# Supplementary material for: Emotional learning promotes perceptual predictions by remodeling stimulus representation in visual cortex
Source: Sci Rep. 2019 Nov 14;9:16867. doi: 10.1038/s41598-019-52615-6 (PMC6856165; doi:10.1038/s41598-019-52615-6)
Supplement: Supplementary file 1 — Efficiency of the conditioning session [file 41598_2019_52615_MOESM1_ESM.pdf]

Emotional learning promotes perceptual predictions by remodeling stimulus representation in visual cortex

Meaux E., Sterpenich, V., Vuilleumier, P.

SUPPLEMENTARY INFORMATION

Behavioral Slowing during subsequent Recognition of the Fear-Conditioned Face

Behavioral performance was virtually flawless across all sessions (familiarization, conditioning, and repetition priming phases). During the main repetition priming experiment, subjects performed an incidental face orientation judgment task in order to engage attention to all stimuli. Behavioral data showed high accuracy across all trial types (mean %correct ± s.d. = 82.1% ± 6.5) with no significant difference between conditions (t=0.51, p=0.62). Response times (RTs) however were reliably influenced by the past emotional history of faces (fig. S1c). A repeated-measures ANOVA of the RT data was conducted using EMOTIONAL HISTORY (i.e. conditioning) (2; famCS+, fam CS-) and REPETITION (2; same, different) as within-subject factor, with Greenhouse-Geisser correction and Newman–Keuls tests for post hoc comparisons. Results showed that RTs were significantly slower to the famCS+ face compared to famCS- (main effect of conditioning, $F(2,42)= 3.41$, $p= .042$, ANOVA). Moreover, this effect was mainly driven by trials when the ID of the famCS+ faces was repeated across the two successive images, unlike the famCS- faces (conditioning×repetition interaction, $F(2,42)= 3.68$, $p=.033$, ANOVA). These findings accord with behavioral slowing (e.g., freezing or attentional capture) typically elicited by the onset of threat stimuli [72,73] and further indicate that previous aversive conditioning induced a lasting change in the perceptual processing of famCS+ faces, persisting during the critical repetition priming session.

Enhanced Brain Responses to Emotionally–Relevant Faces during Aversive Conditioning

To ensure that aversive conditioning impacted on subsequent perception, we performed a whole-brain analysis of fMRI data acquired during the conditioning session. For individual analysis (first level), trial onsets from famCS+, famCS-, and filler faces conditions (fig. S1ab) were modeled in the design matrix as separate regressors, convolved with the canonical HRF function. To account for movement-related variance, the first-level GLM also included realignments parameters from each session [x, y, and z translations and pitch, roll, and yaw rotations] and their temporal derivatives as covariates of no interest. Low-frequency signal drifts were filtered using a cutoff period of 128 s. After model estimation, contrast images were calculated for each experimental condition (vs baseline). The resulting individual maps of t-statistics were then used for the group (second-level) random-effect analyses. Whole-brain statistical maps were computed using a flexible factorial design [70], under the assumption of unequal variance between subjects. Contrast images were computed between famCS+ and famCS- faces to assess the short-term effect of aversive conditioning on face perception. In a second analysis, habituation effects were examined by modeling time as a parametric regressor in the previous GLM for the first level analysis of each individual participant. This procedure allowed us to

isolate brain regions where condition-specific activity negatively correlated with time over the session (i.e., progressive decrease in neural responses).

As expected, comparing the *famCS+ > famCS-* trials showed greater responses in primary auditory cortices (superior temporal gyrus (STG) and Heschl's gyrus), posterior and anterior insula (pINS, aINS), inferior frontal gyrus (IFG), and upper brainstem including PAG (table S1, fig. S1a). These activations are consistent with responses to salient auditory threats that distinguished target faces in the famCS+ condition. We also performed a parametric analysis where time was included as a linear regressor to model habituation over the conditioning session. This analysis revealed a selective condition x time interaction in bilateral fusiform gyrus (FG) and amygdala (AMY): both regions showed higher responses to famCS+ faces that progressively decreased with time during the session, while no time-dependent change occurred in amygdala response for famCS- (table S1, fig. S1b). This results replicate previous studies of aversive face conditioning [74] and therefore confirm that the processing of CS+ face was altered by their negative emotional significance gained during this session.

Supplementary figures and tables captions

Fig. S1|Aversive Conditioning Effects. (a) Brain activity showed differential responses to emotionally-relevant face during the aversive conditioning session, with significant increases for the contrast famCS+ vs. famCS- (see also table 1). **(b)** Habituation over the conditioning session (parametric analysis). Bilateral fusiform gyrus (FG) and amygdala (AMY) activity show higher responses to famCS+ faces that progressively decreased with time. (c) Reaction time as a function of face repetition during the priming session. Aversive conditioning induces slower behavioral responses during subsequent face processing for the famCS+ trials with repeated ID.

Fig. S2| (a) Functional-anatomical segregation for face ID representation within face-selective brain regions. Emotional history-related increases of activity are represented in red (priming session), repetition-related decreases as a function of emotional history in blue (priming session), and face-selective responses (faces > non faces) from an independent localizer scan in yellow. Brain regions in orange indicate activity reflecting both emotional history and face-selective effects, whereas brain regions in green indicate overlaps between face-selective responses and repetition x history interaction effects. Whole brain maps show overlaps between face selective FFA, OFA (top maps) and AMY (bottom maps) as defined by the localizer scan, as well as history and interaction effects observed within FG and IOG and ATL during the repetition priming session. **(b) Results from ROI analyses.** Plots show activity estimates (beta in arbitrary units) for famCS+, famCS-, and new faces as a function of face ID repetition. Data are taken from individual GLM in the priming session, extracted from face-selective FFA, OFA, and AMY in the right hemisphere identified in the face localizer session for each

participant (in yellow, see also suppl table2). The FFA proper shows a main effect of emotional history but no view-independent repetition suppression effects, whereas the OFA shows repetition suppression for the famCS+ condition as opposed to repetition enhancement for the famCS- but no main effect of history. The face-selective AMY cluster shows both a main effect of emotional history and a repetition x history interaction.

Supplementary figures and tables

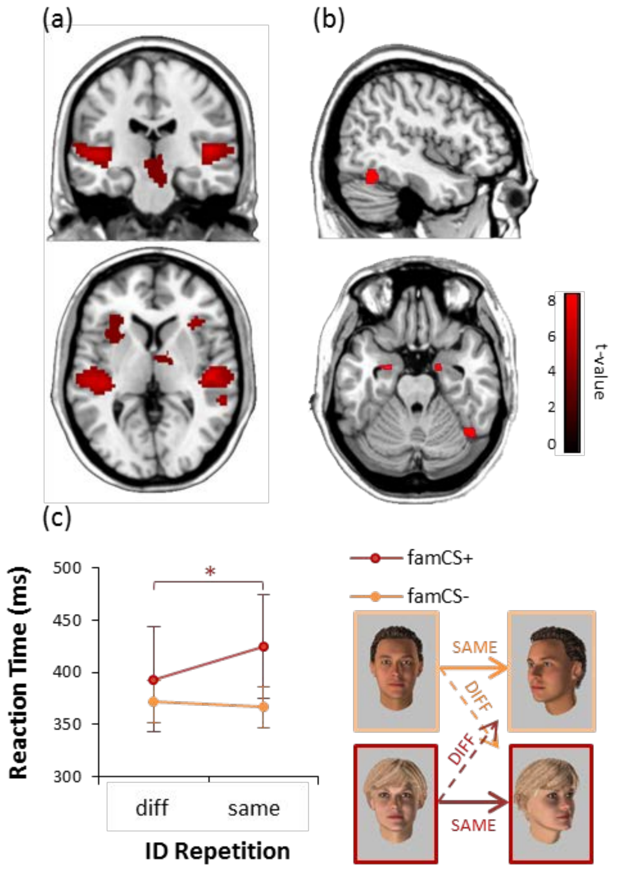

Fig. S1| Aversive Conditioning Effects. (a) Brain activity showed differential responses to emotionally-relevant face during the aversive conditioning session, with significant increases for the contrast famCS+ vs. famCS- (see also table 1). **(b)** Habituation over the conditioning session (parametric analysis). Bilateral fusiform gyrus (FG) and amygdala (AMY) activity show higher responses to famCS+ faces that progressively decreased with time. (c) Reaction time as a function of face repetition during the priming session. Aversive conditioning induces slower behavioral responses during subsequent face processing for the famCS+ trials with repeated ID. *N.B. Faces used in the figure are home-made avatars created using FACsGen software for illustrative purpose only.* Karolinska Face dataset [66] *was used in the experiment.*

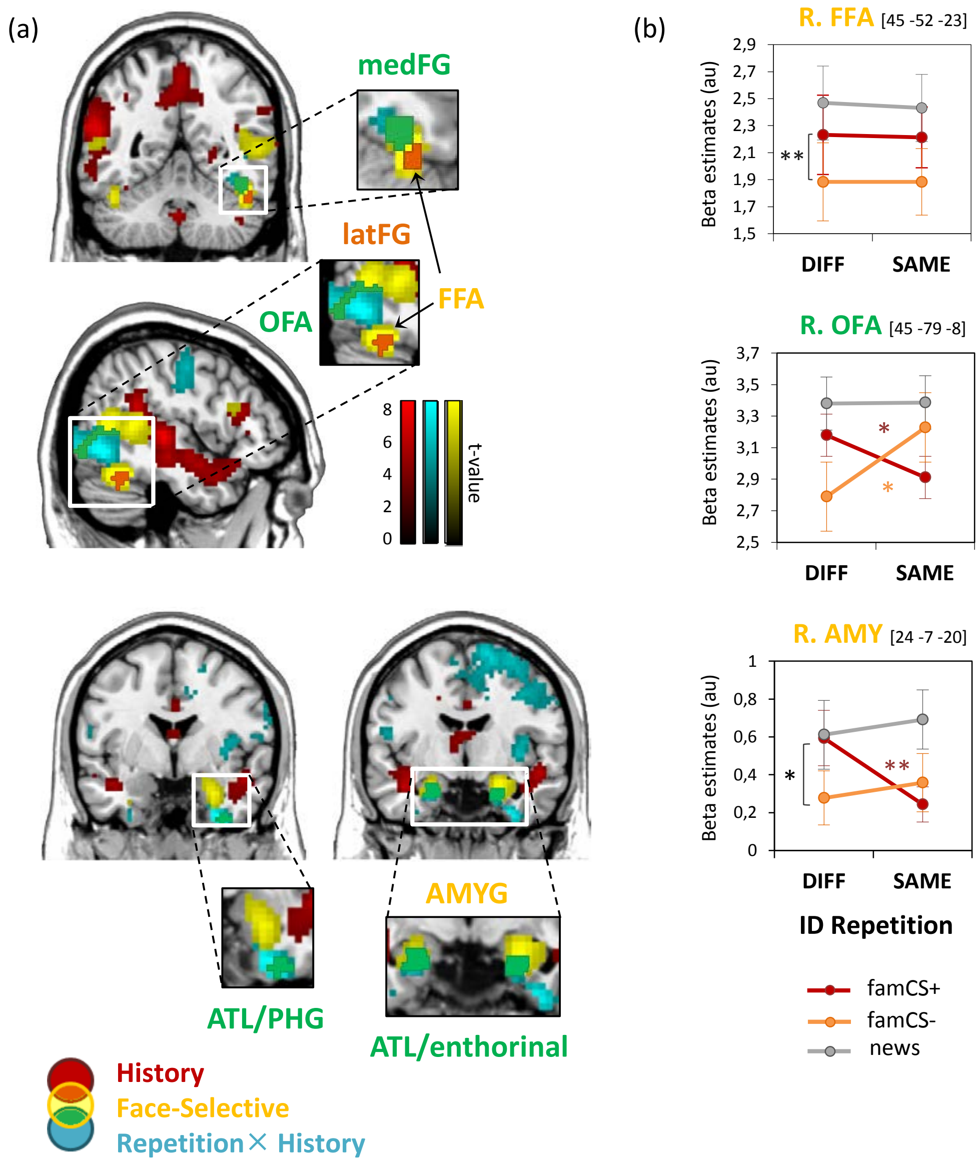

Fig. S2| (a) Functional-anatomical segregation for face ID representation within face-selective brain regions. Emotional history-related increases of activity are represented in red (priming session), repetition-related decreases as a function of emotional history in blue (priming session), and face-selective responses (faces > non faces) from an independent localizer scan in yellow. Brain regions in orange indicate activity reflecting both emotional history and face-selective effects, whereas brain regions in green indicate overlaps between face-selective responses and repetition x history interaction effects. Whole brain maps show overlaps between face selective FFA, OFA (top maps) and AMY (bottom maps) as defined by the localizer scan, as well as history and interaction effects observed within FG and IOG and ATL during the repetition priming session. **(b) Results from ROI analyses.** Plots show activity estimates (beta in arbitrary units) for famCS+, famCS-, and new faces as a function of face ID repetition. Data are taken from individual GLM in the priming session, extracted from face-selective FFA, OFA, and AMY in the right hemisphere identified in the face localizer session for each participant (in yellow, see also suppl table2). The FFA proper shows a main effect of emotional history but no view-independent repetition suppression effects, whereas the OFA shows repetition suppression for the famCS+ condition as opposed to repetition enhancement for the famCS- but no main effect of history. The face-selective AMY cluster shows both a main effect of emotional history and a repetition x history interaction.

	Side	Structures	MNI coords x	y	z	t	Cluster Size
**Supplementary TABLE 1 - BRAIN ACTIVITY DURING CONDITIONING**							
**famCS+ > famCS-** [main effect]	R.	STG	48	-19	4	8.68	333*
	R.	pSTS	51	-31	10	7.39	-
	L.	pINS	-42	-25	1	8.31	377*
	L.	Heschl's G.	-36	-31	10	7.71	-
	L.	STG	-55	-34	16	6.49	-
	R.	aINS/IFG	36	26	4	6.27	47*
			42	32	-5	5.65	-
	L.	aINS/IFG	-27	14	-11	4.95	221#
			-39	14	-2	4.84	-
			-30	26	4	4.35	-
	R/L	Pulvinar	3	-28	8	5.57	310 #
		PAG	3	-31	-20	5.41	-
	R.	pCG	9	-22	31	5.14	51 #
**famCS+ negatively modulated by time [parametric contrast]**	L.	AMY	-27	-7	-20	3.28	37 ~
	R.	AMY	18	-7	-23	3.43	51 ~
	R.	FG	45	-58	-23	4.63	78 ~
	L.	FG	-54	-55	-17	4.89	48 ~

*p < 0.05 FWR corrected at the peak level for the whole brain (random-effect analysis), p < 0.05 ~ Small Volume Corrected (SVC), # p < 0.001 uncorrected

Table S1| Brain activity that differentiates emotionally–relevant face during aversive conditioning. Results from whole-brain analyses on fMRI comparing the *famCS+ > famCS-* trials during the conditioning session (top) and activations for famCS+ faces only that were negatively modulated by time (bottom) (see also fig.3). Coordinates (MNI space) refer to maximally activated foci: x = distance (mm) to the right (+) or the left (−) of the mid sagittal line; y = distance anterior (+) or posterior (−) to the vertical plane through the anterior commissure; z = distance above (+) or below (−) the inter-commissural line. L and R refer to the left and right hemisphere, respectively. p values and their corrections are indicated by the symbols next to the voxel sizes.

	Side	Structures	MNI coords			t	cluster size
			x	y	z		
**Supplementary TABLE 2 - FACE-LOCALIZER RESULTS**							
**FACES** [fearful + neutral] **> NON-FACES** [houses + oval shapes]	R.	pSTS	54	-64	10	9.76	577*
			54	-43	10	9.59	-
	L.	pSTS	-54	-73	16	6.49	85*
			-54	-52	10	5.87	-
	R.	FFA	45	-52	-23	11.76	144*
	L.	FFA	-45	-49	-26	8.13	36*
	R.	OFA	45	-79	-8	6.55	-
	L.	OFA	-39	-85	-14	4.12	29#
	R.	AMY	24	-7	-20	7.96	141*
	L.	AMY	-24	-10	-20	6.75	61*

*p < 0.05 FWR corrected at the peak level for the whole brain (random-effect analysis), # p < 0.001 uncorrected

Table S2| Face-Localizer Results. Brain activity differentiating between faces (fearful and happy) and non faces (houses and oval) perception. Same conventions for coordinates as suppl. table 1.
